# SH3BP5-driven metabolic-immune crosstalk in DLBCL: a prognostic biomarker and therapeutic target for reshaping immunosuppressive microenvironment

**DOI:** 10.1186/s12967-025-06951-z

**Published:** 2025-09-24

**Authors:** Tong Wu, Yi Yang, Yuan Zong, Jiawen Zhao, Xiaoyu Zhao, Lei Li, Yiming Gao, Ning Li, Liting Jiang, Yinyin Xie

**Affiliations:** 1 Department of Stomatology, Ruijin Hospital, Shanghai Jiao Tong University School of Medicine, College of Stomatology, Shanghai Jiao Tong University, Shanghai, 200025 People’s Republic of China; 2 Department of Pathology, Ruijin Hospital, Shanghai Jiao Tong University School of Medicine, Shanghai, 200025 People’s Republic of China; 3 Shanghai Institute of Hematology, State Key Laboratory of Medical Genomics, National Research Center for Translational Medicine at Shanghai, Ruijin HospitalAffiliated to Shanghai Jiao Tong University School of Medicine, Shanghai, 200025 People’s Republic of China

**Keywords:** SH3BP5, Diffuse large b-cell lymphoma (DLBCL), Immune infiltration, Prognostic biomarker, Tumor microenvironment (TME), Oxidative phosphorylation (OXPHOS)

## Abstract

**Background:**

Diffuse large B-cell lymphoma (DLBCL) is a highly heterogeneous and aggressive hematologic malignancy, with the activated B-cell-like (ABC) subtype displaying particularly poor prognosis due to inherent treatment resistance and elevated recurrence rates. Despite advances in targeted therapies and immunotherapies, a significant proportion of patients experience relapse or refractory disease, highlighting the urgent need for novel biomarkers and innovative therapeutic strategies to improve clinical outcomes.

**Methods:**

A multi-dimensional analysis of SH3BP5 expression was performed across DLBCL subtypes, integrating transcriptomic, proteomic, and clinical datasets to assess its correlation with immune infiltration, tumor metabolism, and patient prognosis. Single-cell RNA sequencing data were employed to examine the tumor microenvironment (TME) with higher resolution. Further analysis of the association between SH3BP5 and immune checkpoint gene expression was conducted to explore its potential role in immunotherapy response. Functional in vitro assays were carried out to assess the impact of SH3BP5 knockdown on DLBCL cell proliferation and apoptosis.

**Results:**

The analysis revealed that SH3BP5 is preferentially overexpressed in the ABC subtype of DLBCL across multiple datasets and validated cohorts, and its high expression is significantly associated with poor overall survival. Single-cell transcriptomic profiling demonstrated that SH3BP5 is mainly expressed in malignant B cells and inversely correlated with immune cell infiltration, particularly CD8 + T cells. Mechanistically, pathway enrichment and metabolic assays indicated that SH3BP5 is linked to mitochondrial metabolic reprogramming, promoting oxidative phosphorylation (OXPHOS) and potentially contributing to reduced responsiveness to immune checkpoint inhibitors (ICIs). Functional studies showed that SH3BP5 knockdown significantly suppressed DLBCL cell proliferation, induced apoptosis, and reduced tumor cell viability in vitro.

**Conclusion:**

This study suggests that SH3BP5 may serve as a prognostic biomarker and a potential therapeutic target in DLBCL, particularly within the ABC subtype. By delineating its associations with immune evasion and metabolic reprogramming, these findings provide a mechanistic basis for further exploration of SH3BP5-targeted interventions to help overcome therapy resistance. Future studies in larger clinical cohorts and functional models are warranted to validate these results and assess the potential of integrating SH3BP5 expression profiling into precision medicine strategies for DLBCL.

**Trial registration:**

The study was registered in the Chinese Clinical Trial Registry (ChiCTR2200060430; http://www.chictr.org.cn/) on June 1, 2022.

**Supplementary Information:**

The online version contains supplementary material available at 10.1186/s12967-025-06951-z.

## Introduction

Diffuse large B-cell lymphoma (DLBCL) is the most prevalent form of B-cell non-Hodgkin's lymphoma, marked by substantial clinical and biological heterogeneity [1–3]. Despite therapeutic advancements, both incidence and mortality rates of DLBCL remain high globally, particularly among the elderly [4]. Although DLBCL is potentially curable, approximately 40% of patients remain refractory to treatment, ultimately succumbing to lymphoma recurrence or therapy-related complications [1]. Consequently, alongside efforts to prevent malignant progression, it is crucial to explore the molecular mechanisms behind relapse and dissemination. The identification of novel therapeutic targets and prognostic biomarkers is essential to improving patient outcomes. The emergence of advanced genomic and bioinformatics technologies has significantly aided in uncovering specific metabolic signatures and gene expression patterns, thus creating opportunities for personalized treatment approaches.

DLBCL can be classified into three subtypes based on the cell-of-origin (COO) classification: germinal center B-cell-like DLBCL (GCB-DLBCL), activated B-cell-like DLBCL (ABC-DLBCL), and an unclassified category. These subtypes display distinct clinical and prognostic features, with ABC-DLBCL individuals showing markedly worse survival outcomes [1–3, 5]. The COO classification plays a pivotal role in predicting disease progression and informing treatment strategies. However, studies have highlighted that both the Hans and Choi algorithms provide limited benefits in accurately classifying DLBCL subtypes [6], while traditional gene expression profiling (GEP) remains costly and impractical for routine clinical use [7]. These challenges emphasize the need for ongoing refinement of diagnostic tools to enhance their clinical applicability.

Recent advancements in high-throughput sequencing and single-cell transcriptomics have provided deeper insights into the metabolic heterogeneity of DLBCL. Significant metabolic differences exist among DLBCL subtypes, with studies showing that DLBCL cells can rapidly reprogram their metabolic pathways in response to fluctuations in the tumor microenvironment (TME), adapting to changes in nutrient and oxygen availability. This metabolic flexibility is essential for their survival and progression [8]. Mitochondrial metabolism is particularly critical in certain subtypes, such as OXPHOS-DLBCL, where this metabolic mode enables tumor cells to efficiently utilize mitochondrial nutrients to sustain rapid proliferation and survival. Notably, Caro et al. demonstrated that this OXPHOS metabolic profile in DLBCL is associated with reduced sensitivity to BCR signaling inhibition [9], highlighting a potential interplay between metabolic adaptations and survival pathways in lymphoma development. Consequently, investigating mitochondria-related genes and metabolic alterations is vital for understanding the mechanisms driving tumor development. SH3 Domain-Binding Protein 5 (SH3BP5), also known as SAB, is a mitochondrial protein involved in regulating cellular signaling and metabolic processes [10]. It has been reported to interact directly with Bruton's tyrosine kinase (BTK) and participate in the BCR signaling pathway—one of the critical survival pathways in lymphoma [11]. By modulating the activity of specific enzymes, SH3BP5 is believed to influence ATP synthesis and oxidative phosphorylation, thereby controlling mitochondrial energy supply under various physiological and pathological conditions [12]. Kobayashi et al. reported a correlation between SH3BP5 protein expression and tumor invasiveness in DLBCL [13]. However, the precise role of SH3BP5 in tumor progression and its impact on disease treatment remain unclear, which is the primary focus of this study.

In this study, an integrative bioinformatics approach combined with experimental validation was employed to investigate SH3BP5 expression and its clinical significance in DLBCL. The study examines the role of SH3BP5 in tumor metabolism and its potential implications for disease stratification. Additionally, it proposes a stratified treatment approach based on SH3BP5 expression levels, complementing existing pathological diagnostic panels. These findings contribute to the broader field of precision oncology by offering valuable insights into DLBCL diagnosis, prognosis, and therapeutic strategies, advancing the integration of bioinformatics-driven biomarker discovery into clinical practice.

## Materials and methods

### Clinical sample collection

Tonsil clinical tissue samples utilized in this study were obtained from 20 patients diagnosed with DLBCL at Ruijin Hospital, Shanghai Jiao Tong University School of Medicine. The study was reviewed and approved by the Ethics Committee of Ruijin Hospital and registered with the Chinese Clinical Trial Registry (ChiCTR2200060430).

### Data acquirement and analysis

Five publicly available RNA sequencing datasets of DLBCL were retrieved from the Gene Expression Omnibus (GEO) database, including GSE10846, GSE87371, GSE23501, GSE11318, and GSE182434. The first four datasets comprise bulk RNA transcriptomes. Data normalization was performed using the limma package in R, applying quantile normalization and log2 transformation. GSE182434 is a single-cell dataset consisting of three ABC-DLBCL samples, one GCB-DLBCL sample, and one tonsillitis sample, totaling 18,197 cells.

### Single‑cell RNA‑seq data analysis

Quality control and analysis of the single-cell RNA sequencing data were carried out using the Seurat R package [14]. Cells were retained based on the following criteria: gene count between 300 and 6000, mitochondrial gene percentage < 15%, ribosomal gene percentage > 1%, and hemoglobin gene percentage < 0.1%. After filtering, 18,098 cells remained.

The UMAP algorithm was applied to generate cell clusters. Cluster annotation was performed using the ‘SingleR’ R package, and clusters representing the same cell type were merged based on the expression of known marker genes. Cell–cell communications within the DLBCL microenvironment, particularly among immune cell clusters, were assessed and visualized using the CellChat package.

### Bulk RNA-seq data analysis

Mitochondrial gene expression data were obtained from the MitoCarta databases [15]. Differentially expressed genes (DEGs) were identified using the 'limma' R package, with thresholds of absolute log2|fold-change|≥ 1 and Benjamini–Hochberg *p*-value < 0.05. Gene Ontology (GO) and Kyoto Encyclopedia of Genes and Genomes (KEGG) pathway analyses were conducted using the ClusterProfiler R package. Hazard ratios (HR), 95% confidence intervals, and *p*-values were visualized using the Forest Plot R package. Kaplan–Meier survival curves were generated to assess the relationship between SH3BP5 expression and overall survival (OS) using the survival and survminer packages.

For Gene Set Enrichment Analysis (GSEA), gene signatures from the Hallmark and KEGG gene sets were downloaded from the Molecular Signature Database at GSEA [16]. GSEA was performed with default parameters, utilizing 1000 permutations and considering pathways with p-value < 0.05 and FDR q-value < 0.25 as significant. The WGCNA R package was used to identify highly correlated gene modules through correlation analysis, network construction, and hierarchical clustering. Mitochondrial hub genes were identified by intersecting significant WGCNA module genes with those in the Mitocarta 3.0 database. GO term enrichment was carried out using Metascape.

Genes associated with the oxidative respiratory chain complex were retrieved from the Mitocarta 3.0 database [15], and those related to glycolysis were obtained from the GSEA database. Gene expression in DLBCL and non-cancerous tissues, as well as in tissues with high and low SH3BP5 expression, was visualized using heatmaps. Data analysis was performed with the Limma package, and visualizations were generated using the pheatmap R package. Correlations between selected genes and those involved in the TME were assessed using the Mantel test and Pearson’s correlation coefficient. Interactions were visualized with the ggcor R package (v0.9.8.1).

The immunological characteristics of the TME in DLBCL include the expression of immune modulators, the activity of the cancer immune cycle, immune cell infiltration levels, and the expression of inhibitory immune checkpoints. The stromal, immune, and ESTIMATE scores for DLBCL samples were computed using the ESTIMATE R package [17]. To estimate the proportions of innate and adaptive immune cell types within DLBCL samples, the CIBERSORT and single-sample gene set enrichment analysis (ssGSEA) algorithms were employed [18]. Correlations between immune cell abundance and hub gene expression were analyzed using Spearman’s correlation and visualized with the 'pheatmap' package.

Immune checkpoint-related genes, including PDCD1LG2, CD274, CTLA-4, HAVCR2, PDCD1, and LAG3, were selected, and their expression values were extracted. The circlize R package was used to visualize correlations between SH3BP5 and these immune checkpoint genes. SubMap analysis was conducted via GenePattern to evaluate responses to anti-PD-1 and anti-CTLA-4 immunotherapy based on SH3BP5 expression levels in patients with DLBCL [19].

### Histological staining

Tonsil tissues were fixed in 4% neutral buffered formaldehyde overnight, followed by paraffin embedding and sectioning into 5-μm slices for histological and immunohistochemical analyses. Hematoxylin and eosin (HE) staining and immunohistochemistry (IHC) were performed according to standardized protocols provided by the manufacturer. IHC included deparaffinization, rehydration, antigen retrieval, and blocking. Primary antibodies (listed in Supplementary Table 1) were applied to sections and incubated at 25 °C for 20 min. After PBS washes, secondary antibodies were applied, and sections were developed with 3,3′-diaminobenzidine (DAB, K5007, DAKO, Denmark) as the chromogen. Hematoxylin was used for nuclear counterstaining. Finally, stained sections were analyzed with the BOND Polymer Refine Detection Kit (DS9800, Leica Biosystems) on a Leica Bond RX automated staining system.

### Cell culture, siRNA transfection, and functional analysis

The U-2932 human DLBCL cell line was cultured to mid-logarithmic phase, reaching a high viability (> 90%). Cells were transfected with siRNA from GenePharma using the 4D-Nucleofector system (LONZA, Switzerland) according to the manufacturer’s protocol. After transfection, cells were incubated for further cultivation. The siRNA sequences were: (1) siRNA-SH3BP5-1 sense: GAGAACUGGAGAAGUUAAATT; antisense: UUUAACUUCUCCAGUUCUCTT; (2) siRNA-SH3BP5-2 sense: CCGAAAUCCUGCCGCCUGCTT; antisense: GCAGGCGGCAGGAUUUCGGTT.

Total RNA was extracted from SH3BP5 knockdown strains (48 h post-transfection) and control U-2932 cells using TRIzol reagent (Sigma-Aldrich). Reverse transcription was performed using the HiScript 1st Strand cDNA Synthesis Kit (Vazyme). Quantitative PCR (qRT-PCR) was conducted with SYBR Green (Bio-Rad) on a Bio-Rad CFX96 PCR machine, using glyceraldehyde-3-phosphate dehydrogenase (GAPDH) as the internal control. The primer sequences used for qPCR are as follows: SH3BP5, Forward: TGGCAAAGCTGTGGAAGACTCC, Reverse: TCTGGAAGTCCTGCGTGGCTTT; GAPDH, Forward: GTCTCCTCTGACTTCAACAGCG, Reverse: ACCACCCTGTTGCTGTAGCCAA.

Cell proliferation was measured using the Cell Counting Kit-8 (Beyotime, China). At 72 h post-transfection, CCK-8 solution was added to the medium, and absorbance at 450 nm was recorded after 1 h to assess cell number. Apoptosis was analyzed by flow cytometry. Cells were stained with APC-Annexin-V and PI in Cell Binding Buffer (BD, USA) for 30 min at 4 °C, and analyzed with an LSRFortess™ flow cytometer (BD, USA).

### PCNA and TUNEL immunofluorescence detection

Cell smears were prepared on slides and air-dried briefly before undergoing a 20-min permeabilization treatment. For PCNA detection, samples were incubated with a primary antibody (PCNA, GB12010, Servicebio), followed by incubation with a secondary antibody conjugated to Alexa Fluor 488 (goat anti-rabbit IgG, GB25303, Servicebio). TUNEL staining involved applying a reaction mixture containing TdT enzyme, dUTP, and buffer in a 1:5:50 ratio to the designated area, followed by incubation at 37 °C for 2 h. Both staining protocols included counterstaining with DAPI (10 min at room temperature in the dark), followed by three PBS washes (5 min each). Finally, slides were mounted with an anti-fade medium. Fluorescent images were captured with the following excitation/emission (ex/em) parameters: DAPI (330–380 nm / 420 nm, blue), PCNA (Alexa Fluor 488: 465–495 nm / 515–555 nm, green), and TUNEL (Cy3: 510–560 nm / 590 nm, red).

### Statistical analysis

Statistical analysis was performed using Student’s t-test, one-way ANOVA, or the Chi-square test in GraphPad Prism. Quantitative data are presented as mean ± standard deviation (SD). A *p*-value < 0.05 was considered statistically significant.

## Results

### Mitochondrial-related gene SH3BP5 is overexpressed in ABC-DLBCL and associated with poor prognosis

**Fig. 1:**
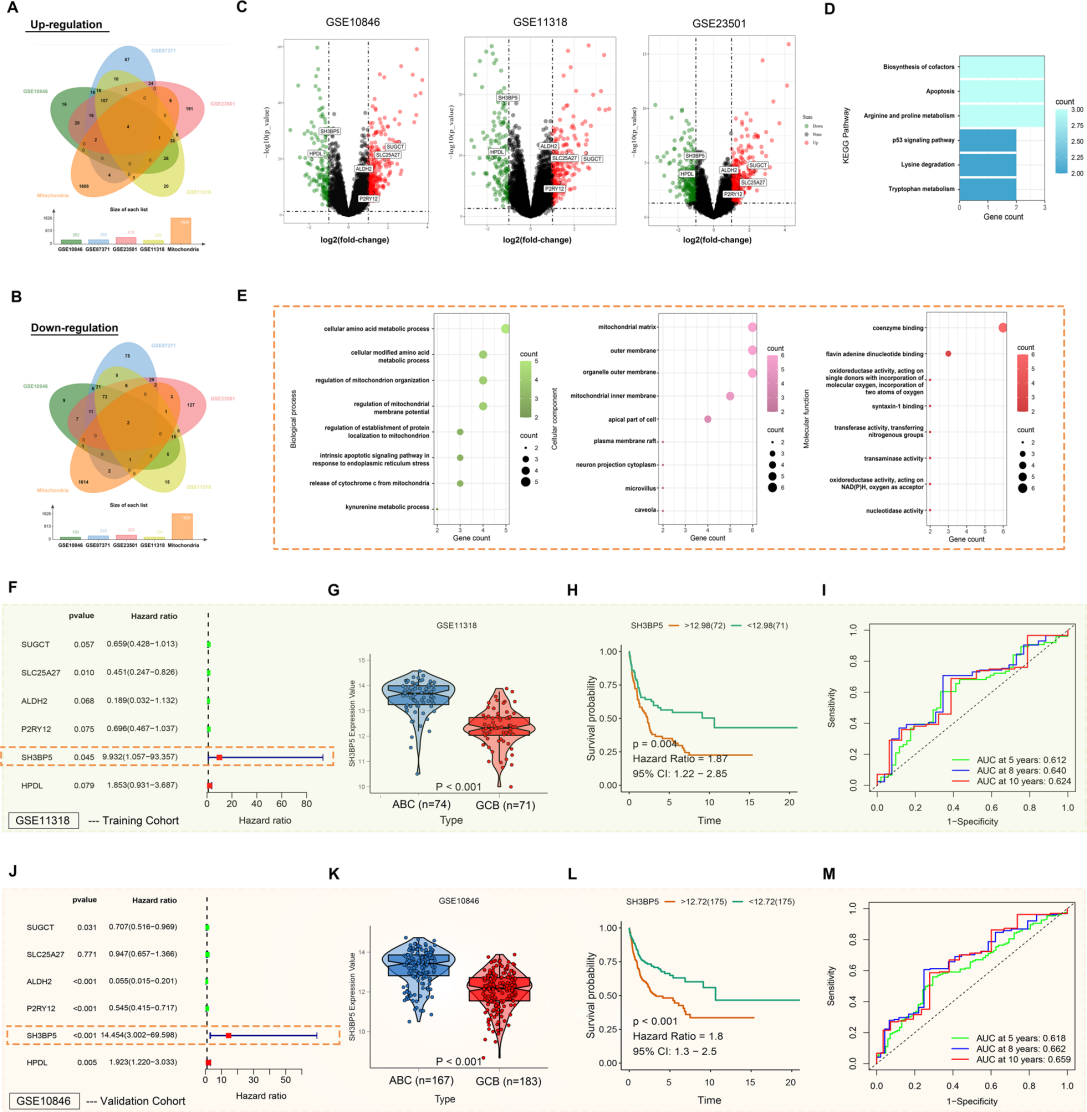
**A** Venn diagram illustrating the overlap between upregulated differentially expressed genes (DEGs) in ABC-DLBCL (GSE10846, GSE87371, GSE23501, GSE11318) and mitochondrial genes from the MitoMiner database. **B** Intersection of downregulated DEGs in ABC-DLBCL and mitochondrial genes. **C** Volcano plots showing DEGs across three cohorts (GSE10846, GSE11318, GSE23501), with red/green dots indicating upregulated/downregulated genes. **D** KEGG pathway enrichment analysis of six hub mitochondrial genes. **E** Gene Ontology (GO) terms enrichment analysis of six hub mitochondrial genes. **F**–**I** Prognostic analysis in the training cohort (GSE11318): **F** univariate cox regression hazard ratios (HR) for hub genes. **G** SH3BP5 mRNA expression in ABC- vs. GCB-DLBCL (*p* < 0.001). **H** Kaplan–Meier survival curves stratified by SH3BP5 expression (high vs. low, log-rank *p* = 0.004). **I** Receiver operating characteristic (ROC) curve of SH3BP5 for survival prediction (AUC = 0.612/0.640/0.624 at 5/8/10 years). **J**–**M** Independent validation in the GSE10846 cohort (analyses parallel to **F**–**I**)

To identify DEGs between patients with the ABC subtype and GCB subtype of DLBCL, data from the GEO database, including GSE10846, GSE87371, GSE23501, and GSE11318, were analyzed (Fig. 1C). By intersecting mitochondrial-related genes with DEGs upregulated in the ABC subtype (Fig. 1A), four genes were identified. Similarly, by intersecting with downregulated DEGs (Fig. 1B), two additional genes were found. These six differentially expressed mitochondrial-related genes (SUGCT, SLC25A27, ALDH2, P2RY12, SH3BP5, and HPDL) were defined as hub genes and subjected to KEGG (Fig. 1D) and GO (Fig. 1E) pathway enrichment analyses. The results indicated that these hub genes are primarily involved in mitochondrial metabolic functions and cellular amino acid metabolism, including redox reactions, amino acid transport, and apoptosis. These genes also play significant roles in key cellular structures, such as cell and mitochondrial membranes, and are associated with various metabolic pathways, including amino acid metabolism, p53 signaling, and tryptophan metabolism. The GSE11318 samples were used as a training group for univariate Cox analysis, which led to the selection of SH3BP5 as the final gene (Fig. 1F). Additionally, SH3BP5 expression was significantly higher in the ABC group compared to the GCB group in the GSE11318 dataset (Fig. 1G). A Kaplan–Meier plot was generated by dividing the samples into high- and low-SH3BP5 groups based on the median expression level (12.98), revealing that the low-SH3BP5 group had a better prognosis (Fig. 1H). Further validation with ROC curve analysis confirmed the predictive value of SH3BP5 (Fig. 1I). Validation in the GSE10846 dataset (Fig. 1J-M) demonstrated high consistency with the initial findings, reinforcing the robustness of our conclusion.

### WGCNA identifies hub genes associated with high expression of SH3BP5

**Fig. 2:**
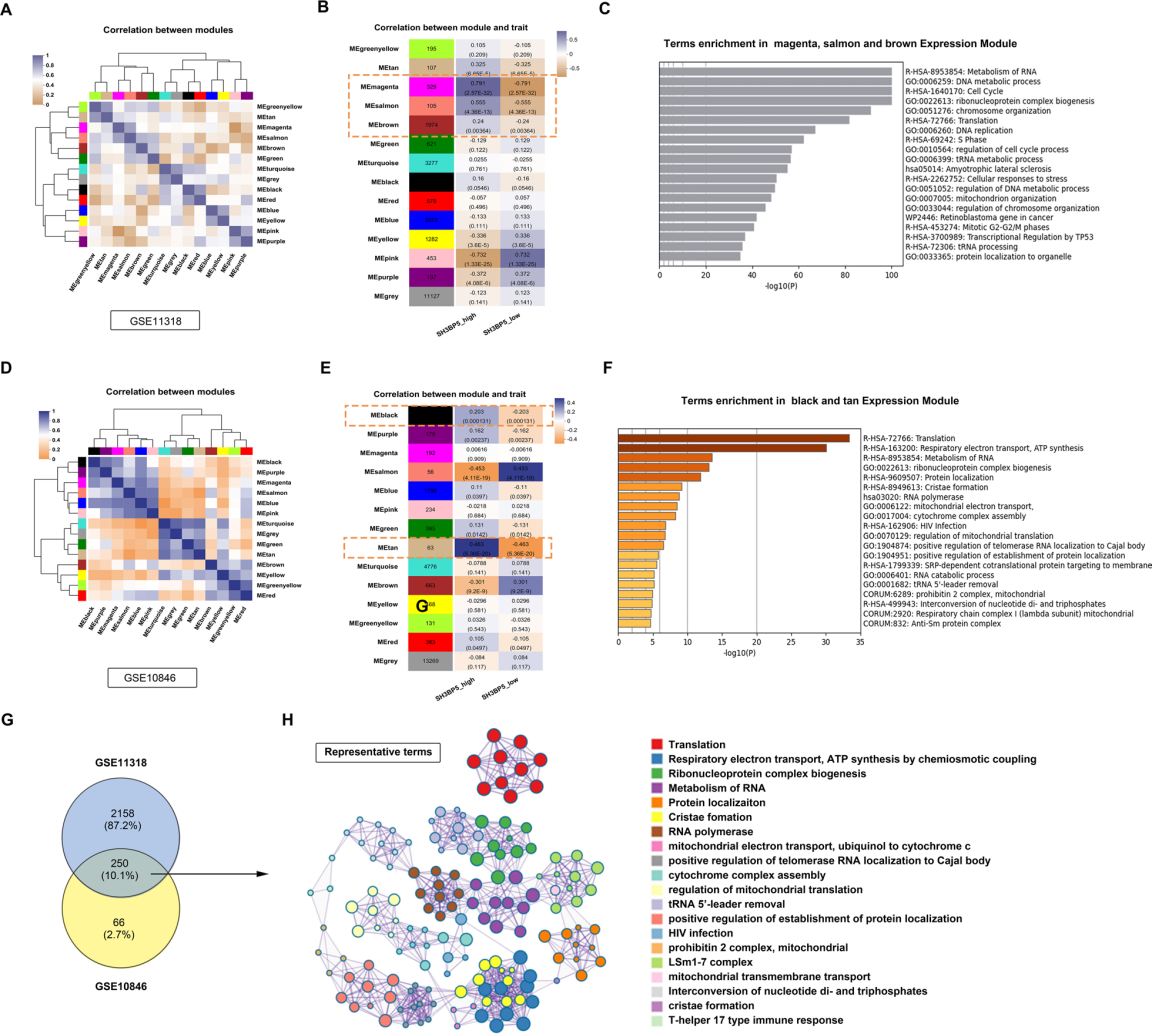
**A**–**C** Training cohort (GSE11318): **A** Hierarchical clustering of 14 distinct co-expression modules (color-coded) identified by WGCNA. **B** Module-trait relationship heatmap correlating gene modules with DLBCL subtypes. Purple/orange denote positive/negative correlations, with correlation coefficients and *p*-values indicated. **C** Top enriched Gene Ontology (GO) terms (Metascape) for key modules (magenta, salmon, brown) linked to mitochondrial metabolism. **D**–**F** Validation cohort (GSE10846): **D**–**F** Parallel WGCNA module clustering, trait correlation, and functional enrichment analyses (as in **A**–**C**). **G** Venn diagram identifying 250 consensus genes overlapping between metabolic-immune modules (magenta/salmon/brown in GSE11318; black/tan in GSE10846). **H** Functional annotation network of consensus genes, with node colors representing enriched GO clusters (Metascape). Core pathways include respiratory electron transport (blue) and RNA metabolism (purple)

Given the pivotal role of SH3BP5 in DLBCL classification and prognosis, WGCNA was performed on the GSE11318 training dataset, resulting in 14 major co-expression modules (Fig. 2A). The magenta, salmon, and brown modules were identified as key modules (Fig. 2B). Metascape analysis based on GO terms revealed that these modules are predominantly enriched in processes related to RNA and DNA metabolism, as well as cell cycle regulation (Fig. 2C). Similar results were obtained using GSE10846 as a validation set (Fig. 2D–F). The analysis of 250 common genes from the magenta, salmon, and brown modules in GSE11318 and the black and tan modules in GSE10846 (Fig. 2G) showed enrichment in mitochondrial function, RNA metabolism, protein synthesis, and localization (Fig. 2H), suggesting their critical role in cellular energy metabolism, gene expression regulation, and protein function.

### SH3BP5 expression is linked to tumor microenvironment and metabolic pathway gene enrichment

**Fig. 3:**
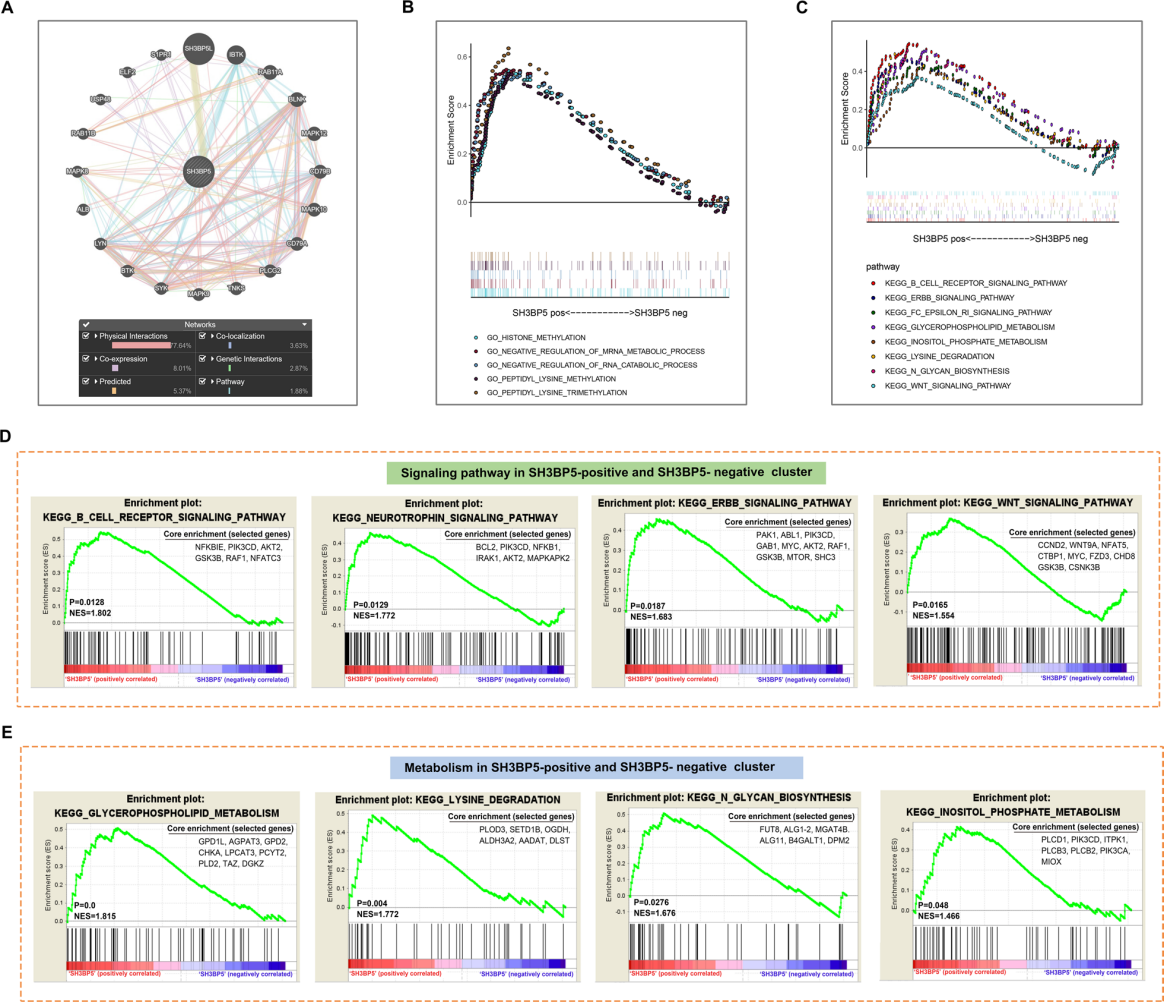
**A** Genes interacting with SH3BP5 were predicted using GENEMANIA. **B**–**E** Data from GSE10846, grouped according to SH3BP5 correlation: **B** GO biological processes enrichment of SH3BP5-correlated genes. **C** KEGG pathway enrichment of SH3BP5-correlated genes. **D**–**E** Gene set enrichment analysis (GSEA) comparing SH3BP5-positive vs. negative correlation groups: **D** GSEA reveals that gene sets related to BCR, neurotrophin, ERBB, and WNT pathways are predominantly enriched in the SH3BP5 positively correlated gene group. **E** GSEA reveals that gene sets associated with glycerophospholipid metabolism, lysine degradation, N-glycan biosynthesis, and inositol phosphate metabolism are predominantly enriched in the SH3BP5 positively correlated gene group

To elucidate SH3BP5’s functional network, its interacting partners were identified using GeneMANIA, revealing significant associations with mitochondrial regulators (e.g., S1PR1 and MAPK8/9/10) and immune modulators (e.g., CD79A) (Fig. 3A) [20]. GO/KEGG analyses of SH3BP5-correlated genes further demonstrated its regulatory capacity, with positively correlated genes enriched in histone modification, RNA splicing, and metabolic pathways critical for mitochondrial membrane dynamics and energy homeostasis (Fig. 3B-C). Among these, we focused on pathways of particular interest to our study: GSEA analysis revealed that the SH3BP5-positive cluster showed significant enrichment in gene sets associated with BCR and WNT signaling pathways (Fig. 3D), as well as metabolic pathways, including glycerophospholipid metabolism (mitochondrial membrane biogenesis), lysine degradation (acetyl-CoA generation), N-glycan biosynthesis (protein glycosylation), and inositol phosphate metabolism (calcium homeostasis) (Fig. 3E). These pathways collectively highlight SH3BP5’s role in driving metabolic reprogramming towards OXPHOS dependency, a characteristic feature of the OXPHOS-DLBCL subtype. The coordinated upregulation of lipid remodeling and glycosylation pathways further suggests that SH3BP5 orchestrates mitochondrial structural integrity to sustain energy production, while lysine degradation may fuel TCA cycle flux to meet the bioenergetic demands of aggressive ABC-DLBCL.

### SH3BP5 is predominantly expressed in B cells and is positively correlated with mitochondrial OXPHOS metabolism

**Fig. 4:**
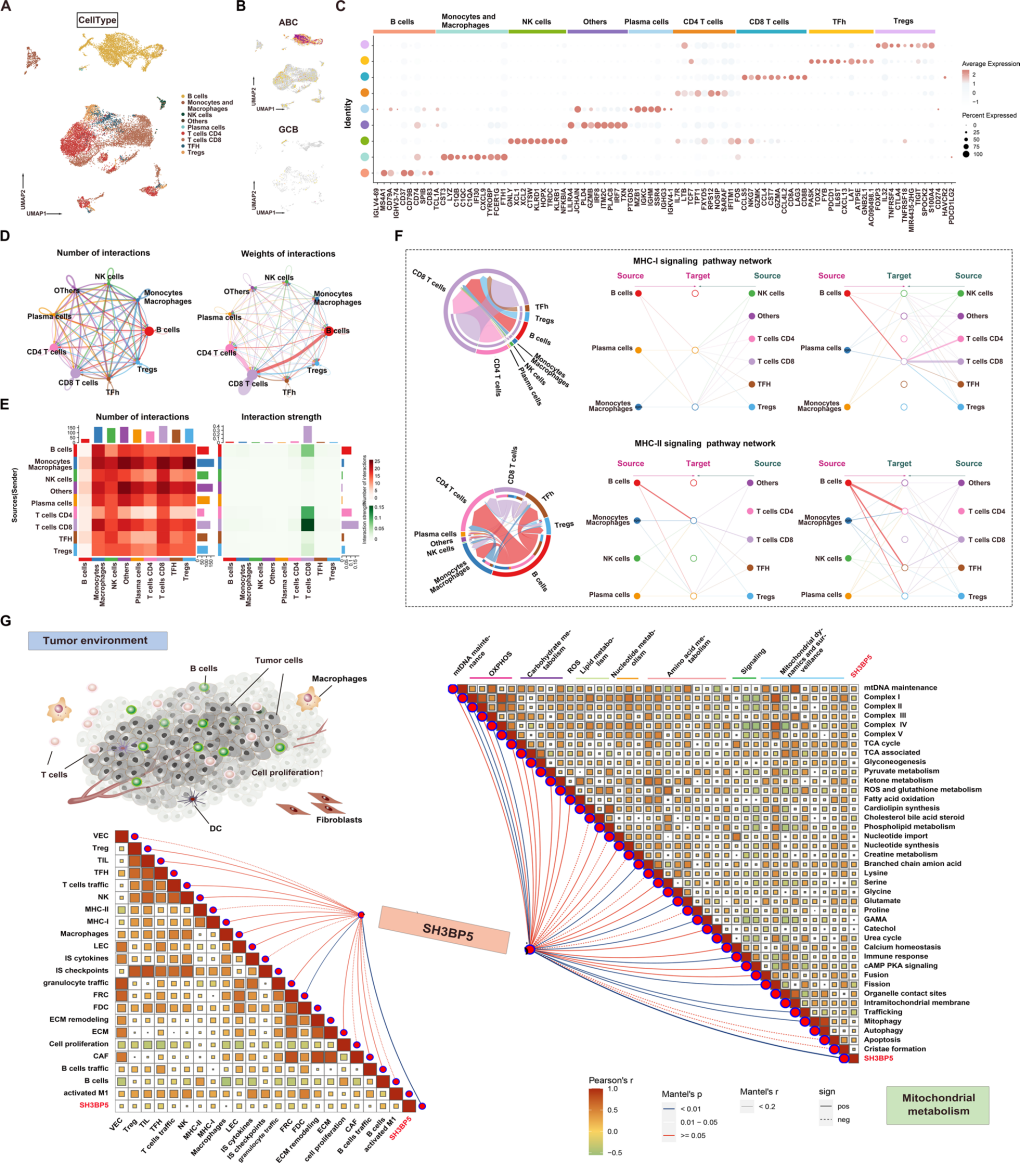
**A** Uniform Manifold Approximation and Projection (UMAP) of 9 major cell clusters across 4 DLBCL tumors and 1 tonsillitis control. Cell types are annotated using canonical markers (refer to panel C). **B** UMAP plots showing the expression of SH3BP5 in ABC-DLBCL and GCB-DLBCL subtypes. **C** Expression of marker genes used to annotate the nine major cell types. **D**–**E** Circle plot and heatmap illustrating differences in the number and strength of interactions between various cell clusters. **F** Chord diagram and hierarchical plot displaying interactions of MHC-I and MHC-II signaling pathways between different cell clusters. **G** Correlation matrix between SH3BP5 and mitochondrial metabolism genes (MitoCarta3.0). Colors represent Pearson’s correlation coefficient (r) between SH3BP5 and mitochondrial metabolism-related genes, with red indicating a positive correlation (Pearson’s r > 0) and green indicating a negative correlation (Pearson’s r < 0). Statistical analysis was performed using the Mantel test, with the blue line indicating a *p*-value < 0.01 and the white line indicating 0.01 < *p* < 0.05

Single-cell transcriptomic analysis (GSE182434) provided a comprehensive view of the cellular architecture of DLBCL tumors, identifying B cells, CD8 + T cells, and CD4 + T cells as the dominant immune populations (Fig. 4A). A dot plot further demonstrated the expression of canonical marker genes across these cell subsets (Fig. 4C). SH3BP5 expression was predominantly localized to malignant B cells (Fig. 4B). Intercellular communication analysis revealed strong MHC-I/II signaling between SH3BP5-high B cells and CD8 + T cells (Fig. 4D-F), suggesting a potential role for SH3BP5 in modulating antigen presentation and T cell exhaustion. Notably, SH3BP5 expression was positively correlated with follicular helper T cells (TFH) and fibroblastic reticular cells (FRC/FDC) (Fig. 4G). Additionally, SH3BP5 expression positively correlated with mitochondrial OXPHOS components (e.g., Complexes I, II, IV, and V) and regulators of mitochondrial dynamics (such as cardiolipin synthesis, fission, organelle contact sites, and mitophagy) (Fig. 4G), further reinforcing its dual role in supporting both the immunosuppressive TME and the bioenergetic demands of OXPHOS-dependent DLBCL.

### SH3BP5 high expression correlates with immunosuppressive microenvironment in DLBCL

**Fig. 5:**
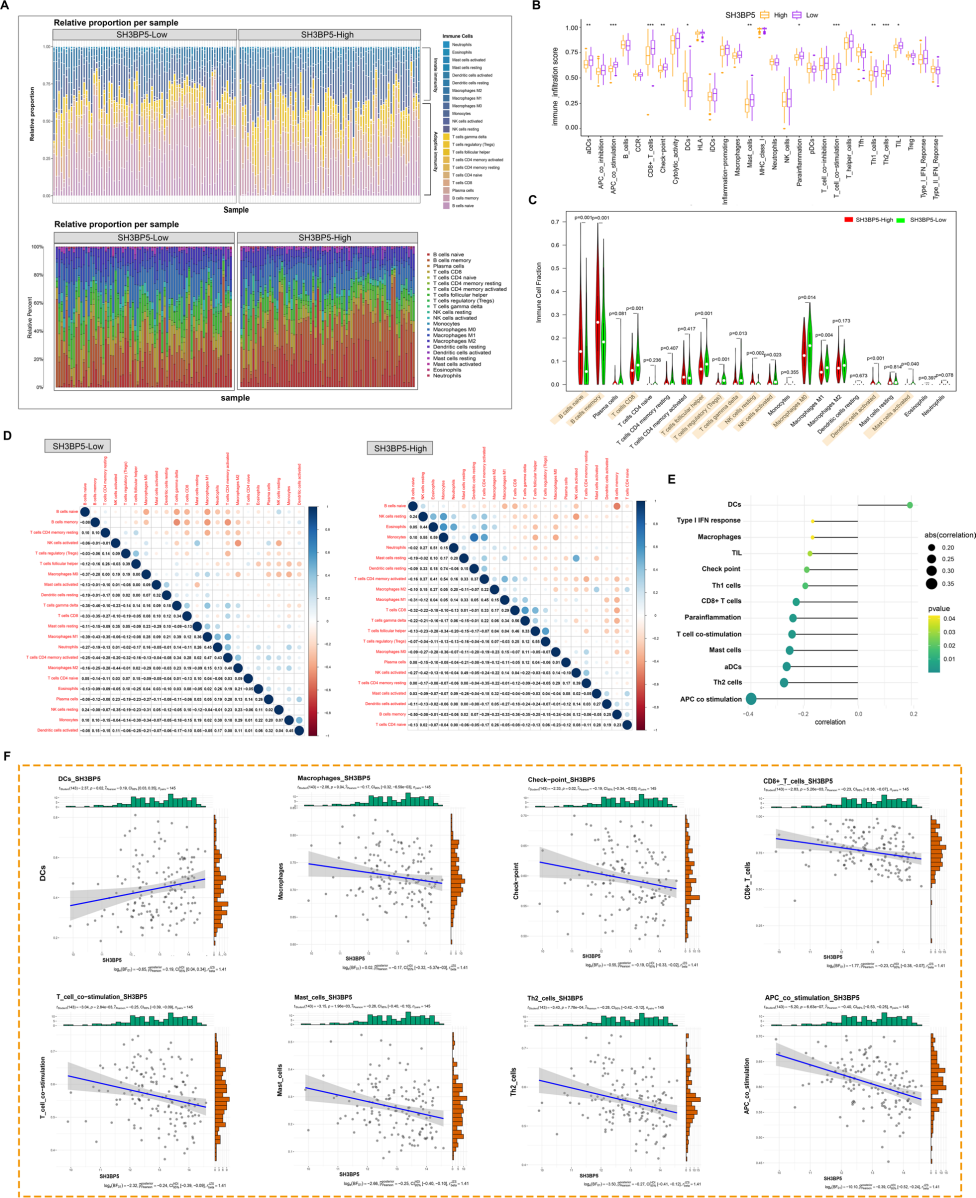
**A** CIBERSORT quantification of innate vs. adaptive immune cell infiltration in GCB- and ABC-DLBCL subtypes (GSE11318 cohort). **B**–**C** Abundance of various immune cells in high- and low-SH3BP5 groups based on the ssGSEA and CIBERSORT algorithms. **D** Pearson correlation among different immune cells in high- and low-SH3BP5 groups using the CIBERSORT algorithm. **E** Lollipop graph showing the correlation of SH3BP5 with immune cells. The size of the lollipop represents the strength of the correlation, while the lollipop color represents the *p*-value. **F** Scatterplots validating key associations: DCs, macrophages, checkpoint molecules, CD8 + T cells, T cell co-stimulation, Mast cells, Th2 cells, and co-stimulatory molecules

To elucidate SH3BP5’s impact on the DLBCL immune landscape, systematic deconvolution of bulk RNA-seq data (GSE11318) was performed. CIBERSORT analysis confirmed the dominance of adaptive immune cells in both GCB and ABC subtypes (Fig. 5A), while ssGSEA revealed a significant correlation between high SH3BP5 expression and alterations in the infiltration scores of various immune cell types (Fig. 5B). Comparative CIBERSORT profiling identified 10 immune subsets with significant shifts in abundance between SH3BP5-high and -low groups (*p* < 0.05, Fig. 5C). Furthermore, the correlation patterns of immune cells between high- and low-SH3BP5 expression groups differed notably (Fig. 5D). Specifically, high SH3BP5 expression was negatively correlated with most immune cell subpopulations (Fig. 5E), with significant negative correlations observed for mast cells, macrophages, and T cells, while dendritic cells (DCs) exhibited a positive correlation (Fig. 5F). These results highlight SH3BP5’s potential regulatory role in immune cell distribution and activity.

### SH3BP5 expression as a diagnostic and stratification marker for DLBCL

**Fig. 6:**
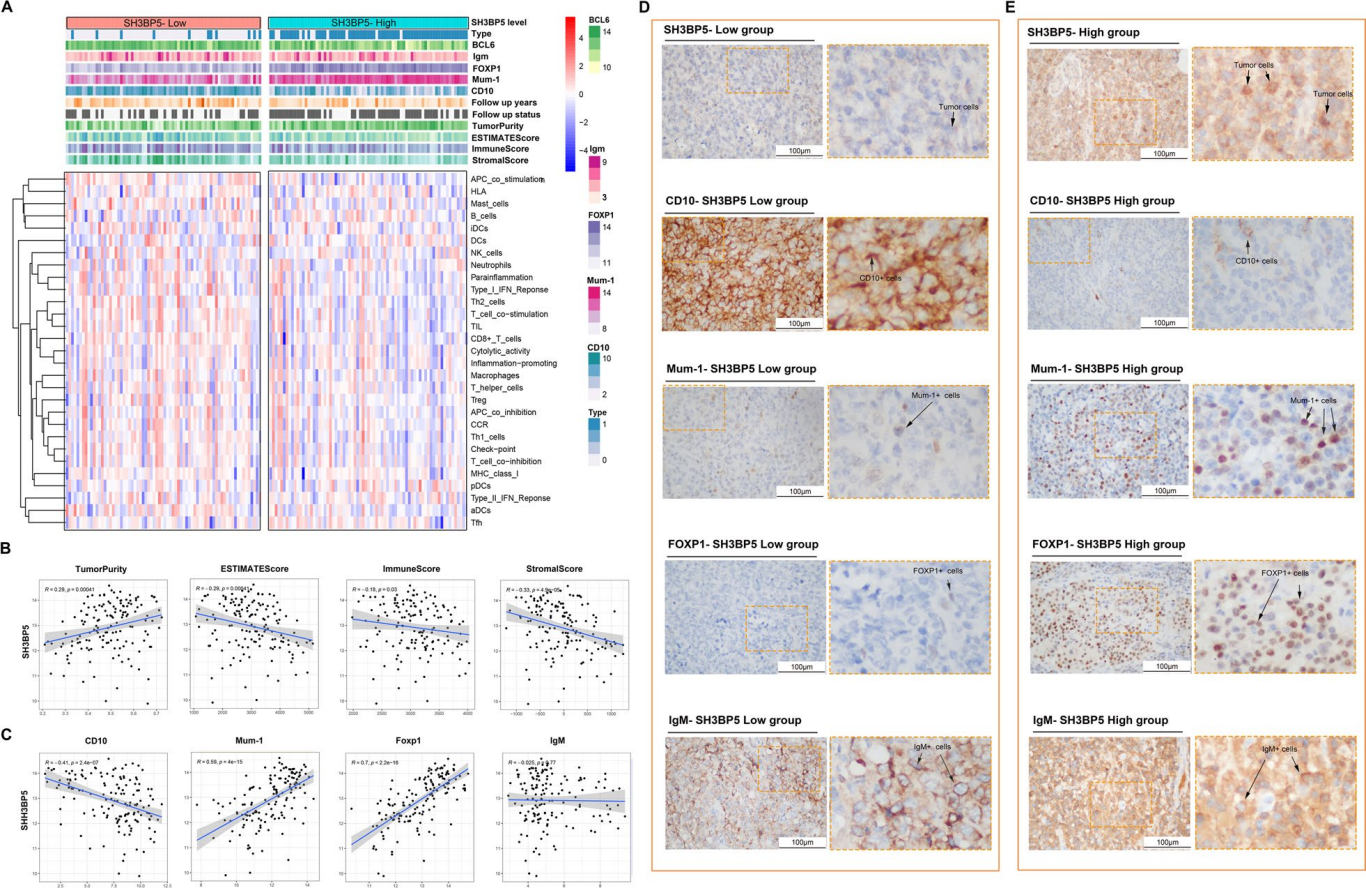
**A** Unsupervised hierarchical clustering of SH3BP5-stratified DLBCL tumors (GSE11318), integrating ssGSEA-derived immune response signatures and ESTIMATE algorithm outputs: tumor purity, immune score, and stromal score. **B** Scatterplots correlating SH3BP5 expression with tumor purity, ESTIMATE score, immune score, and stromal score. **C** SH3BP5 correlation with standard diagnostic markers: positive for ABC-defining markers (MUM1, FOXP1, IgM) and negative for GCB marker CD10. **D**–**E** Representative immunohistochemistry (IHC) images of CD10, MUM1, FOXP1, and IgM in (**D**) SH3BP5-high vs. (**E**) SH3BP5-low tumors. Scale bar: 100 µm

To assess SH3BP5’s diagnostic value, GSE11318 cases were stratified into SH3BP5-high and -low groups, and their TME profiles and canonical subtype markers were compared (Fig. 6A). SH3BP5-high tumors exhibited reduced stromal/immune scores and increased tumor purity (Fig. 6B), aligning with its role in suppressing anti-tumor immunity. Theophraste et al. developed and validated a new immunohistochemical algorithm for DLBCL classification based on traditional gene expression profiling (GEP), incorporating one GCB marker (CD10) and three ABC markers (FOXP1, Mum-1, IgM) (see Supplementary Table 2 for details) [21]. SH3BP5 expression positively correlated with ABC-defining markers (FOXP1, Mum-1) and inversely with the GCB marker CD10 (Fig. 6C), mirroring the immunohistochemical classification framework (Fig. 6D-E). Importantly, integrating SH3BP5 into this algorithm improved the accuracy of ABC/GCB discrimination, as it uniquely captures mitochondrial metabolic features (e.g., OXPHOS signature) alongside immunophenotypic criteria.

### SH3BP5 mediates immune suppression through regulation of multiple immunomodulators

**Fig. 7:**
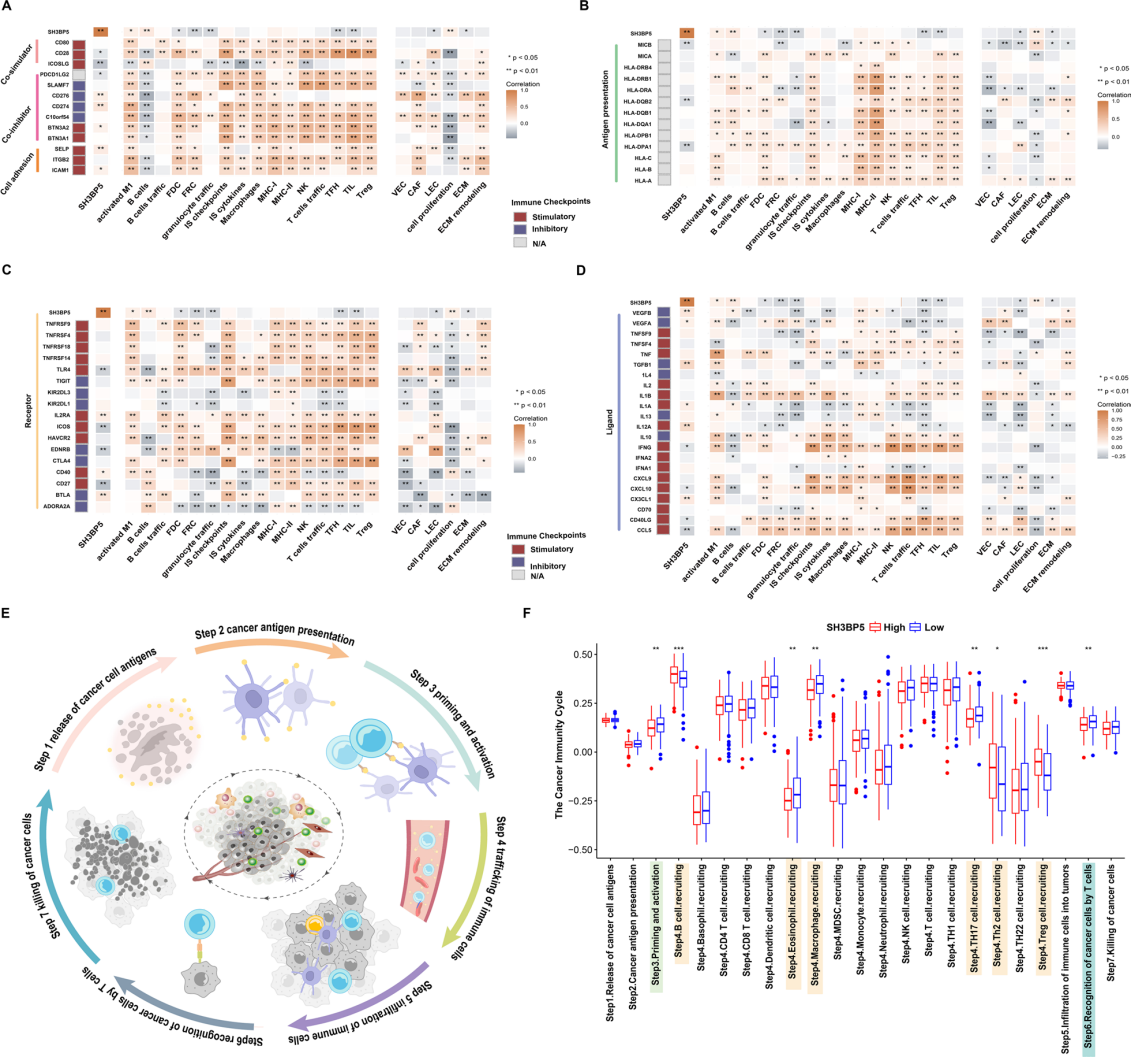
**A**–**D** Heatmaps depicting Pearson correlations between SH3BP5 expression and immunomodulators in DLBCL: **A** Co-stimulators (e.g., CD80, CD28), co-inhibitors (e.g., PDCD1LG2, CD274), and cell adhesion molecules (e.g., SELP, ICAM1). **B** Antigen presentation molecules (e.g., HLA-DRA, MICB). **C** Immune checkpoint receptors (e.g., LAG3, HAVCR2). **D** Ligands (e.g., VEGFB, CX3CL1). **E** Schematic of the cancer-immunity cycle: release of cancer cell antigens (1) → cancer antigen presentation (2) → priming and activation (3) → trafficking of immune cells (4) → infiltration of immune cells (5) → recognition of cancer cells (6) → killing of cancer cells (7). **F** Differences in the various steps of the cancer immunity cycle between high- and low-SH3BP5 groups in the GSE10846 dataset

Building on SH3BP5’s association with immune depletion, its regulatory impact on immunomodulators was further explored. SH3BP5-high tumors exhibited inverse correlations with key checkpoint inhibitors (PDCD1) and T cell co-stimulators (ICOS and CD28) while showing positive associations with B cell markers (CD19 and MS4A1) (Fig. 7A-D). This bidirectional regulation—suppressing cytotoxic immunity while promoting B cell dominance—aligns with its metabolic role in OXPHOS-driven immune evasion. Additionally, SH3BP5 was significantly associated with cell proliferation, suggesting its involvement in regulating tumor cell growth. To functionally validate these associations, the cancer-immunity cycle, which includes antigen presentation, immune cell recruitment, and tumor cell killing [22], was analyzed. SH3BP5-high tumors displayed broad suppression of cycle activity, including priming and activation, immune cell recruitment (e.g., B cells and Treg cells), and the recognition of cancer cells by T cells (*p* < 0.05) (Fig. 7E-F). This suppression suggests that mitochondrial hyperactivity drives tumor growth while restricting anti-tumor immunity.

### Explore potential treatment strategies based on different SH3BP5 expression patterns

**Fig. 8:**
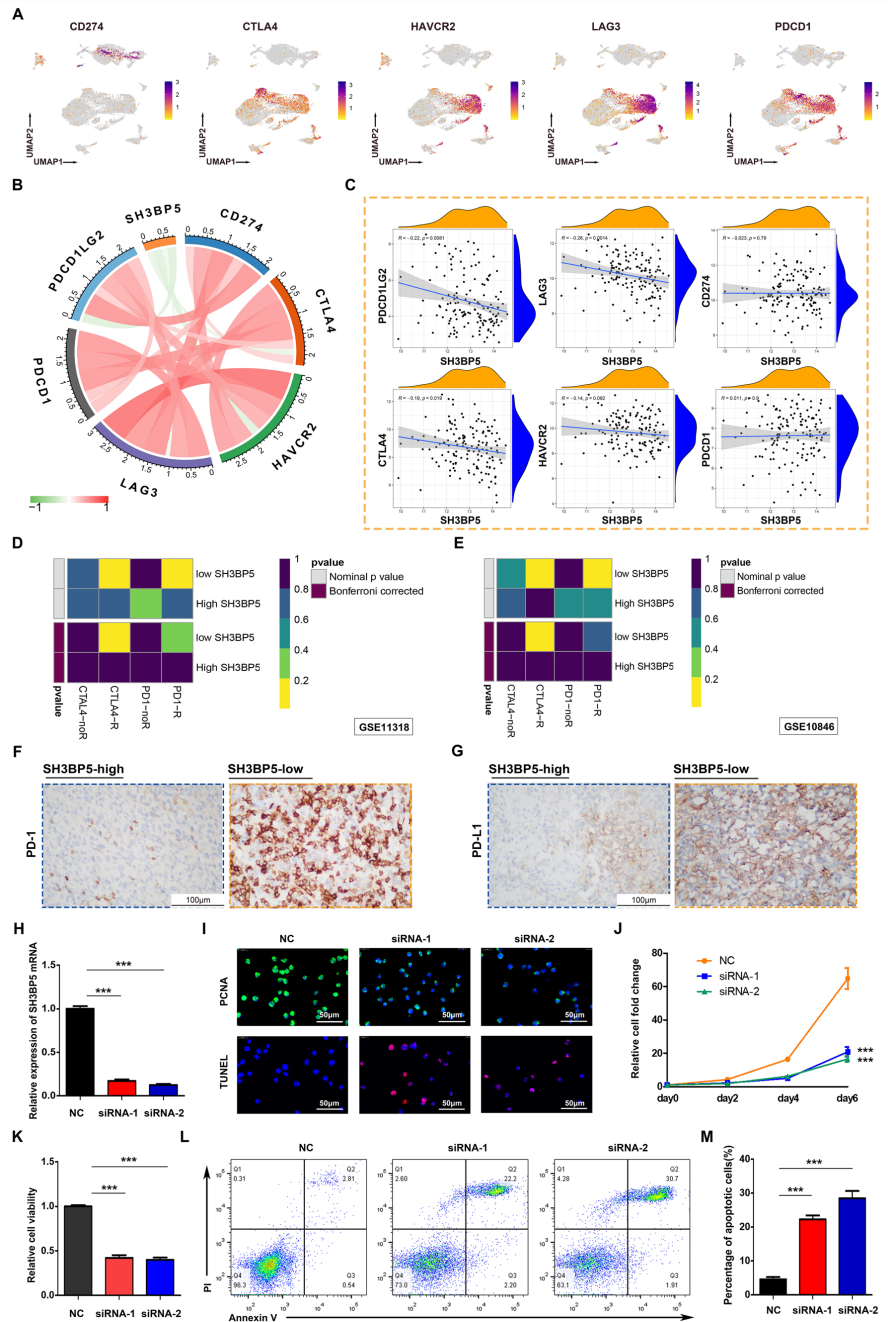
**A** UMAP feature plots demonstrating differential expression of selected classic immune checkpoint genes (PDCD1, PDCD1LG2, CD274, CTLA-4, HAVCR2, and LAG3). **B** Correlation between the expression of SH3BP5 and immune checkpoint molecules in GSE11318 (Red—Positive, Green—Negative). **C** Scatter plot depicting the correlation between SH3BP5 and immune checkpoint gene expression levels. **D**–**E** SubMap analysis of patient response to immunotherapy (CTLA-4 or PD-1 inhibitors) at different expression levels of SH3BP5 in GSE11318 and GSE10846. **F**–**G** IHC validation of PD-1 and PD-L1 downregulation in SH3BP5-high tumors. **H** qRT-PCR analysis confirming SH3BP5 mRNA knockdown in U-2932 human DLBCL cells treated with siRNA. **I** Immunofluorescence showing reduced PCNA (green, proliferation marker) and increased TUNEL (red, apoptosis) in SH3BP5-knockdown cells. **J** Cell counts post-transfection. **K** Comparison of cell viability among NC, siRNA-1, and siRNA-2 groups. **L**–**M** Flow cytometry quantifying apoptosis post-knockdown

Given SH3BP5’s role in immune checkpoint regulation, its potential as a predictive biomarker for immunotherapy response was investigated. Single-cell analysis localized checkpoint molecules (HAVCR2, CTLA-4, PDCD1, LAG3) predominantly to CD8 + T cells (Fig. 8A), with expression levels inversely correlated with SH3BP5 at both the bulk transcriptomic and protein levels (Fig. 8B-C). To explore the impact of SH3BP5 expression on immunotherapy, response rates to therapies targeting cytotoxic T lymphocyte-associated antigen-4 (CTLA-4) and programmed cell death protein 1 (PD-1) were assessed in patients with DLBCL exhibiting high and low SH3BP5 expression using a subclass mapping approach. Notably, patients with low SH3BP5 expression exhibited more favorable responses to anti-PD-1 and anti-CTLA-4 therapies compared to those with high SH3BP5 expression (Fig. 8D-E). Immunohistochemical staining further confirmed a decrease in PD-1 and PD-L1 protein expression in the SH3BP5-high group, reinforcing the association between SH3BP5 and immune checkpoint activity (Fig. 8F-G). These results suggest that SH3BP5 could serve as a potential biomarker for predicting the efficacy of PD-1 and CTLA-4 monoclonal antibody therapies in DLBCL. Finally, the functional role of SH3BP5 in DLBCL cell apoptosis and proliferation was validated through in vitro experiments. SH3BP5 expression was knocked down in U-2932 cells using two independent siRNAs, as confirmed by qRT-PCR (Fig. 8H). SH3BP5 knockdown led to reduced proliferation (PCNA immunofluorescence, Fig. 8I; cell fold change, Fig. 8J) and viability (Fig. 8K), while promoting apoptosis (TUNEL staining, Fig. 8I; flow cytometry, Fig. 8L-M). These results confirm SH3BP5’s dual role in promoting proliferation and inhibiting apoptosis.

## Discussion

Despite decades of extensive research, many patients with DLBCL continue to face recurrence or complications, underscoring a persistent challenge in clinical management [23]. This highlights the critical need to identify novel prognostic biomarkers and therapeutic targets to improve patient survival and treatment outcomes. This study systematically explores the dual biological role of SH3BP5 in ABC-DLBCL through integration of multi-omics analysis and functional validation. Our findings demonstrate that elevated SH3BP5 expression correlates significantly with poor survival outcomes, a relationship consistently observed across multiple independent patient cohorts. Survival analysis, diagnostic performance evaluation, and immunohistochemical validation further substantiate the clinical reliability of SH3BP5 as a robust biomarker for risk stratification.

Given the high heterogeneity of DLBCL, accurate molecular subtype classification is essential for predicting patient prognosis and developing tailored treatment strategies to improve survival and quality of life [24]. Traditional GEP techniques, such as RNA sequencing and qPCR, offer high precision in identifying DLBCL subtypes but are limited by high costs, long processing times, and technical complexity, hindering their widespread clinical use [7, 25]. In contrast, the Hans algorithm, relying on immunohistochemistry with multiple antibody combinations (e.g., CD10, BCL6, MUM1), introduces operational complexity and subjective interpretation [26]. Theophraste et al. proposed a more streamlined IHC-based algorithm incorporating CD10, MUM1, FOXP1, and IgM, demonstrating greater clinical practicality by simplifying the diagnostic process and reducing inter-observer variability [21]. This study not only validates the reliability of this algorithm but also suggests the inclusion of SH3BP5 to further enhance its prognostic power. By leveraging SH3BP5’s unique metabolic-immune regulatory functions—such as its dependence on mitochondrial OXPHOS and its role in modulating the immunosuppressive TME—this integrative approach significantly strengthens the biological relevance of DLBCL subtype classification. This refined strategy has the potential to address the limitations of existing methods, providing a more comprehensive molecular foundation for personalized treatment decisions and improving the prediction of therapeutic responses.

Mitochondria function as critical signaling and metabolic centers in cancer cells, dynamically orchestrating energy production and survival pathways to drive tumor progression [27, 28]. This dual role positions mitochondrial metabolism as a promising therapeutic target, particularly in hematological malignancies such as DLBCL, where translational inhibition strategies are gaining momentum [29]. In DLBCL, dependency on mitochondrial OXPHOS is significantly heightened, reflecting an increased ATP demand necessary for rapid proliferation and metabolic adaptation to microenvironmental stress [30, 31]. SH3BP5, a mitochondrial outer membrane protein, regulates the JNK signaling pathway to maintain a balance between cell survival and apoptosis. Our findings reveal that SH3BP5 is selectively overexpressed in ABC-DLBCL relative to GCB-DLBCL and functions as an independent prognostic marker for poor survival, linking mitochondrial function with clinical outcomes. Furthermore, emerging evidence indicates that SH3BP5 influences tumor stemness by modulating the BCR and WNT signaling pathways, while its interaction with the JNK axis may contribute to resistance to chemotherapy and immunotherapy [32]. These insights align with our study’s findings on the pivotal role of mitochondrial metabolism in DLBCL progression, positioning SH3BP5 as a key metabolic-immune regulator with translational implications.

The metabolic heterogeneity within DLBCL further underscores the therapeutic significance of SH3BP5. Norberg et al. classified DLBCL into OXPHOS-dependent and BCR-dependent subtypes, with the former exhibiting increased mitochondrial pyruvate flux and heightened electron transport chain activity [33]. Our results support this classification: SH3BP5 expression strongly correlates with the activity of OXPHOS complexes (I, II, IV, V), functionally identifying SH3BP5-high tumors as OXPHOS-DLBCL, a subtype that directly influences treatment response. Notably, this subtype demonstrates enhanced sensitivity to mitochondrial translation inhibitors like tigecycline—an antibiotic that targets mitochondrial ribosomes and selectively disrupts OXPHOS-DLBCL energy metabolism [33, 34]. Given SH3BP5’s critical role in maintaining OXPHOS dependency, its expression level may serve as a predictive biomarker to identify patients likely to benefit from tigecycline-based therapies. This "metabolism-immune dual intervention" strategy presents a novel approach to overcoming treatment resistance. SH3BP5-high patients could benefit from a combination of mitochondrial translation inhibitors (e.g., tigecycline) and ICIs (e.g., anti-PD-1 therapy), capitalizing on their synergistic effects on immune microenvironment remodeling. Conversely, SH3BP5-low patients may exhibit a more immunoreactive TME and respond better to ICIs alone. This molecular phenotype-based treatment stratification offers significant promise for improving the precision and efficacy of DLBCL clinical management.

The complex interplay between mitochondrial metabolism and TME remodeling has emerged as a critical axis in DLBCL pathobiology [35]. Our data demonstrate that SH3BP5-high DLBCL tumors exhibit significantly reduced infiltration of anti-tumor immune effectors, such as CD8 + T cells, alongside decreased stromal-immune scores and increased tumor purity. This observation aligns with the emerging understanding that DLBCL heterogeneity arises not only from distinct cell-of-origin subtypes but also from dynamic TME reprogramming, where mitochondrial fitness governs immune cell functionality [36].

In recent years, advances in understanding the role of inflammatory responses in tumor growth and metastasis have provided new insights into the mechanisms of cancer progression [37]. Building upon this, SH3BP5-mediated modulation of the TME extends to the regulation of immune checkpoints—which are components of the immune system that cancer cells exploit to evade immune clearance and are key determinants of immune evasion and therapeutic response [38]—suggesting that alterations in the TME may enhance the efficacy of immune checkpoint inhibitors [39]. While ICIs have transformed cancer treatment, their efficacy in DLBCL remains hindered by primary resistance mechanisms [40, 41]. Our findings indicate that SH3BP5-low tumors, characterized by an immunoreactive TME with increased immune infiltration and upregulated checkpoint molecules, demonstrate superior responses to PD-1 and CTLA-4 blockade compared to SH3BP5-high tumors. This dichotomy suggests a self-reinforcing loop: SH3BP5-driven OXPHOS hyperactivity suppresses immune surveillance, triggering compensatory upregulation of immune checkpoints in immunocompetent tumors, a vulnerability exploitable by ICI therapy.

Mechanistically, SH3BP5 may stabilize this immunosuppressive state via lactate-mediated inhibition of DC maturation and T cell priming, positioning it as a metabolic gatekeeper of immune evasion [42]. This metabolic reprogramming not only promotes tumor cell survival but also fosters an immune escape-permissive environment, underscoring SH3BP5’s dual role in metabolic adaptation and immune modulation. These insights highlight the potential of targeting mitochondrial metabolism and TME interactions as therapeutic strategies in DLBCL, particularly in overcoming ICI resistance and restoring anti-tumor immunity [43]. By elucidating the intricate relationship between mitochondrial function and the immune landscape, this study paves the way for novel therapeutic approaches that exploit the metabolic vulnerabilities of DLBCL tumors and their immunosuppressive microenvironment.

While this study establishes SH3BP5 as a metabolic-immune nexus in DLBCL through multi-omics integration, significant challenges remain in translating these findings into clinically actionable strategies. The upstream molecular pathways through which SH3BP5 regulates immune checkpoints like PD-L1 remain poorly defined. Additionally, our conclusions are primarily derived from retrospective cohorts and in vitro models, necessitating further validation in patient-derived xenograft models to evaluate the in vivo efficacy and safety of targeted interventions. Moreover, real-time monitoring techniques for tracking SH3BP5 dynamic expression during treatment have yet to be developed—an essential advancement for optimizing adaptive therapeutic strategies in precision oncology.

## Conclusion

SH3BP5 emerges as one of a regulator of metabolic-immune crosstalk in ABC-DLBCL, functioning as both a prognostic biomarker and therapeutic target. Its overexpression drives mitochondrial OXPHOS dependency and immune suppression, correlating with poor survival and resistance to immune checkpoint inhibitors. By integrating SH3BP5 into diagnostic algorithms, it may be possible to improve subtype classification and guide treatment decisions more precisely. This work bridges mitochondrial metabolism and immune evasion, providing a novel framework for advancing DLBCL management.

## Supplementary Information

Below is the link to the electronic supplementary material.

Supplementary Material 1

Supplementary Material 2

Supplementary Material 3

Supplementary Material 4

## Data Availability

Public datasets analyzed in this study are available in the Gene Expression Omnibus (GEO) repository: https://www.ncbi.nlm.nih.gov/geo/ (Accession codes: GSE10846, GSE11318, GSE23501, GSE87371, GSE182434). Analysis tools and pipelines: Seurat (v4.0, single-cell RNA-seq analysis): http://satijalab.org/seurat/. ESTIMATE (tumor microenvironment scoring): http://bioinformatics.mdanderson.org/estimate/index.html. CIBERSORT (immune cell deconvolution): http://cibersort.stanford.edu. GenePattern (SubMap analysis): https://cloud.genepattern.org. GeneMANIA (gene interaction networks): https://genemania.org. Specialized databases: MitoCarta (mitochondrial proteomics): http://www.broadinstitute.org/mitocarta. GSEA Molecular Signatures Database (MSigDB): http://software.broadinstitute.org/gsea/msigdb/.
