# Supplementary material for: SH3BP5-driven metabolic-immune crosstalk in DLBCL: a prognostic biomarker and therapeutic target for reshaping immunosuppressive microenvironment
Source: J Transl Med. 2025 Sep 24;23:1003. doi: 10.1186/s12967-025-06951-z (PMC12462315; doi:10.1186/s12967-025-06951-z)
Supplement: Supplementary file 1 — Supplementary Material 1 [file 12967_2025_6951_MOESM1_ESM.docx]

**Supplementary Table 1**

**Antibody Reagents and Specifications for DLBCL Immunohistochemistry**

| Target | Catalog Number | Dilution | Company |
| --- | --- | --- | --- |
| SH3BP5 | 11127-2-AP | 1：100 | Proteintech |
| CD10 | IR64861 | Ready-to-use | Dako Denmark A/S |
| Mum-1 | GA64461 | Ready-to-use | Dako Denmark A/S |
| PD-1 | ZM-0381 | Ready-to-use | Beijing Zhongshan Golden Bridge Biotechnology |
| PD-L1 | SK00621 | Ready-to-use | Dako Denmark A/S |
| CD19 | ZM-0038 | Ready-to-use | Beijing Zhongshan Golden Bridge Biotechnology |
| Foxp1 | ab134055 | 1：350 | abcam |
| IgM | IR513 | Ready-to-use | Dako Denmark A/S |
