# Supplementary material for: SH3BP5-driven metabolic-immune crosstalk in DLBCL: a prognostic biomarker and therapeutic target for reshaping immunosuppressive microenvironment
Source: J Transl Med. 2025 Sep 24;23:1003. doi: 10.1186/s12967-025-06951-z (PMC12462315; doi:10.1186/s12967-025-06951-z)
Supplement: Supplementary file 2 — Supplementary Material 2 [file 12967_2025_6951_MOESM2_ESM.docx]

**Supplementary Table 2**

**Algorithm based on one GCB marker (CD10) and three ABC markers (MUM1, FOXP1 and IgM)**

| Algorithm | Classification |
| --- | --- |
| CD10 positive AND one (or more) ABC marker negative | GCB |
| CD10 negative AND two (or more) ABC markers positive | ABC |
| CD10 negative AND two (or more) ABC markers negative | GCB |
| All the other combinations | Unclassified |
